# 肺癌加速康复外科体系的建立及优化

**DOI:** 10.3779/j.issn.1009-3419.2017.12.01

**Published:** 2017-12-20

**Authors:** 国卫 车

**Affiliations:** 610041 成都，四川大学华西医院胸外科 Department of Thoracic Surgery, West China Hospital, Sichuan University, Chengdu 610041, China

**Keywords:** 加速康复外科, 加速肺康复, 肺外科, 肺肿瘤, Enhanced recovery after surgery, Enhanced lung recovery after surgery, Lung surgery, Lung neoplasms

## Abstract

加速康复外科（enhanced recovery after surgery, ERAS）理念应用于不同疾病及学科均有其相应的关键技术及流程与体系。肺癌微创外科的核心是加速肺康复（enhanced lung recovery after surgery, ELRAS），而加速肺康复的关键技术是气道管理和肺保护。气道管理和肺保护的实现需要医、护、康一体及多学科协作，从而形成肺癌患者加速肺康复的完整体系。主要包括以下几方面：一是ERAS方案实施的各个环节均有准确、客观的评估体系；二是ERAS方案简单、易行且具有可重复性；三是ERAS方案临床应用效果具有精准严谨评价体系；四是以问题为导向的团队架构。总之，加速肺康复外科体系需要在临床实践中不断优化与完善。

## Abstract (en)

With the development of key techniques and programs, the concept of enhanced recovery after surgery (ERAS) has been utilized in a wide range of diseases and surgical specialties. The key technological elements of enhanced lung recovery after surgery (ELRAS), which is regarded as the mainstay of minimally invasive lung cancer surgery, consist of the airway management and the lung protection. A multidisciplinary team cooperation based on physicians, nurses and rehabilitative therapists is needed to achieve the goals of airway management and lung protection, thus establishing a comprehensive ELRAS system for lung cancer patients. Such ELRAS systems should meet the following demands: firstly, there are accurate and objective assessment models for each section in an ELRAS program; secondly, the ELRAS protocols should be made as simple and repeatable as possible; thirdly, there are rigorous evaluation systems for the clinical efficacy of an ELRAS program; finally, a question-oriented team should be constructed in an ELRAS program. In summary, the ELRAS system needs to be constantly optimized and improved in the clinical practices.

加速康复外科（enhanced recovery after surgery, ERAS）"no pain and no risk"实现需要多学科协作且不断优化围手术期流程^[1]^。围手术期流程优化体现在^[2]^：一是术前合理评估及强调对高危因素的预防（如肺栓塞及并存疾病）；二是术中流程优化，如麻醉（无气管插管及尿管），手术微创等；三是术后加强症状管理，如疼痛，管道和饮食等。围手术期加速肺康复方案各个环节的准确、完整和有效的完成，才能使患者获益。而贯穿围手术期的所有流程构成了加速肺康复外科体系，即简单易行可重复的临床应用方案、客观准确的评估与评价体系和合理的团队架构^[3]^。探讨肺癌加速康复外科（enhanced lung recovery after surgery, ELRAS）顺利实施的途径，旨在为实现加速肺康复外科的规范化、有效性提供参考。

## 1 ERAS评估体系

ERAS评估体系是实行肺癌患者"个体化或精准化"治疗的前提，采用适合于特定人群的合理方案需要准确评估。客观和准确的评估才能保证ERAS方案的顺利、有效的执行^[4]^。

ERAS评估体系主要包括：术前评估和每一个实施环节（方案）评估体系，术前评估主要是将患者进行分类（如"正常"、"高危"和"症状"人群），并采取相应ERAS方案^[5]^；而环节或过程评估主要是指对某特定人群所采取的统一流程中，每个流程是否都需要实施，实施的方案是什么等，如ERAS方案中对"正常"人群的优化流程中的管道管理：尿管是否安置，若不安置，患者术前宣教需要做什么，术后护理工作重点是什么等^[6, 7]^；再如术后胸腔引流管的应用、一根或两根、管径大小、留置位置等^[8-10]^。其他如高危因素的评估，如男性且吸烟患者，除戒烟外，还需要加强气道管理及排痰，而女性不吸烟患者则主要改善气道高反应性缓解气道痉挛^[11-13]^。症状患者的评估有助于围手术期的精准处理，如咳嗽、疼痛和气短等^[14, 15]^。我们目前针对肺癌患者采用的评估体系主要包括：入院宣教及术前评估系列：胸部疾病调查问卷、肺癌患者心晴指数问卷、肺栓塞风险评估表和围手术期肺功能评估；术中评估系列：如尿管应用评估表、胸腔引流管应用评估表、手术器械包评估表和镇痛方案评估。术后评估系列：术后饮食及中药应用评估表、症状评估表、咳嗽程度评估、气短评估表、疼痛满意度调查表、切口管理评估表及随访评估表等。

## 2 ERAS方案体系

不同的肺癌患者因其病史、伴随疾病和肿瘤严重程度不同，决定了他们不能应用统一的ERAS方案^[16]^。如何准确促进患者的加速肺康复，需要"个体化"和"可重复"的ERAS方案。"个体化"的方案主要强调方案的可操作性，肺癌需要手术的患者通过术前的评估可以分为三类：①"正常"患者；②"症状"患者；③"高危因素"患者。"可重复”的方案主要是指方案本身可推广，结合我国情况主要是指方案的每个环节要有可替代方案和2个-3个备选方案，以确保各单位根据自身情况选择性应用。

### 2.1 "正常"肺癌患者的ERAS方案

"正常"肺癌患者是指即年龄小于60岁，且体检发现的小结节，无明显伴随疾病的患者。这类患者的ERAS方案是以微创技术的合理应用为核心，优化手术相关流程，以缩短平均住院日或日间手术能否运用作为评价标准。可优化的流程主要有：麻醉是否需要气管插管（根据医院情况而定）；尿管留置是不必的^[6, 7]^；两根胸腔引流管或单根粗引流管（管径大于28 F）是不必要的，用细引流管（小于24 F）或不用引流管是可取的^[8-10]^；术后镇痛可用肋间神经阻滞或口服甾体类止痛药，对胃肠功能影响大的药物是不可取的^[17]^。管道及镇痛的合理应用，可以使患者早期下床活动并促进胃肠功能快速恢复^[18]^。

### 2.2 “症状”肺癌患者的ERAS方案

“症状”肺癌患者是指术前患者具有临床症状或出院后发生的与手术相关的常见症状（如咳嗽、气短和疼痛等）^[14]^，以控制症状为主的术前治疗措施或基于术后症状而对手术技术或过程改进或优化为关键，以改善患者生活质量为核心。肺癌患者术前合并症状的治疗不但有助于降低术后并发症，也有助于提高住院舒适度和术后生活质量。研究表明，胸腔镜肺癌患者术后常见症状依次为咳嗽、疼痛和气短。术后病理证实Ⅰ期肺癌患者，术前仍有52.5%的患者以咳嗽为主诉就诊^[19]^。而肺癌患者出院后导致咳嗽的危险因素为术前有咳嗽症状和麻醉时间长^[20, 21]^。通过围手术期的肺康复训练，可以有效降低肺癌患者术后咳嗽的严重程度，没有降低咳嗽的发生率，需要优化围手术期肺康复训练方案。胸腔镜肺癌患者术后疼痛主要部位为胸腔引流管口，疼痛性质为胀疼，考虑主要原因为引流管粗需缝线固定或引流管拔出后的预置线结扎导致，缓解或降低疼痛发生主要是改进胸腔引流的相关措施，而不是过分依赖镇痛药^[17]^。疲劳与气短也是影响肺癌患者术后生活质量改善的主要因素，术后发生气短的肺癌患者与肿瘤分期晚、手术范围大，及肺功能相对差有关，围手术期相应的肺功能训练，可以有效降低术后气短的发生程度^[14]^。

### 2.3 肺癌患者合并高危因素的ERAS方案

"高危因素"患者主要是指因患者自身或医疗相关因素导致围手术期并发症或死亡率增加^[11]^。自身因素主要是指合并伴随疾病（如慢性阻塞性肺疾病、血压等），而伴随疾病主要与年龄、生活习惯（如吸烟等）相关；医疗因素主要与手术过程相关，如麻醉时间过长（包括手术时间长）、手术创伤（术中肺挫裂伤、失血或输液过多等），而外科因素常常因各种原因被忽视^[4]^。肺癌合并高危因素患者的ERAS方案的核心是肺康复训练，同时医疗因素是优化流程和加强管理。

肺康复训练方案不但要简单、易行且训练时间不能太长。主要包括药物康复和物理康复；药物康复以袪除气道炎症和缓解支气管痉挛为主，目的是通畅气道^[12, 13, 22, 23]^：包括抗生素、祛痰药和消炎药或平喘药的应用，要按照药物说明书及临床规范应用，但有些药物的说明书的用法与用量均是从治疗疾病出发，目前尚缺乏外科应用指征及剂量，需要临床研究获得证据。物理康复主要是肢体肌肉及呼吸肌训练，以提高运动及呼吸耐力，改善呼吸功能为主^[22-25]^：适合于胸外科的主要包括激励式肺量计吸气训练、功率自行车运动训练和登楼梯训练（可选其中一个）。肺康复训练时间以3 d、7 d、14 d作为参考^[24-27]^。

## 3 ERAS评价体系

ERAS临床应用方案实施效果的准确评价不但保障患者术后顺利康复，也是优化方案和流程的客观依据。ERAS方案应用的总体评价，是否达到应有的临床效果，需要从以下3个方面评价：一是围手术期并发症降低和住院时间缩短、患者症状改善及生活质量提高和社会满意度提高及治疗费用降低^[28-30]^。二是ERAS方案实施每个环节的实时评价，保证每个环节实施的有效性。三是ERAS方案的实施需要多学科协作，包括很多连续执行的流程，任何一个环节的失误或无效都会导致整个ERAS方案的失败或效果不理想。ERAS执行过程中每个环节合理评价，才可确保"承上启下"交接的有效性，最终保障整个方案的有效运行。同时每个环节的正确评价也有助于整个流程的优化。

ERAS评价体系的核心是需要客观的评价指标。ERAS目前总体评价指标客观性强，易于对比，如平均住院日、并发症降低发生率和住院总费用及社会满意度等。但是这些指标有时不能准确反映是否与采取了ERAS方案有关或与ERAS方案的某个环节有关，因为外科理论、技术及管理的改进也会改善这些指标。而目前ERAS评价体系中每个流程或环节实施的评价指标仍然缺乏，这正是临床应用ERAS困难与困惑所在。如肺癌需要肺康复训练的患者，方案制订好了，不管训练场合（康复科、呼吸科、社区医院或家庭），如何评价达到手术要求呢？目前通用或大家能够接受的指标是一秒用力呼气容积（forced expiratory volume in one second, FEV_1_）的改善程度或达到手术需要的最小值。可是现有的研究表明，短期肺康复训练改善FEV_1_的程度较小，表明FEV_1_可能不是评价肺康复训练的最有效或最好的指标，但它可能是改善慢性阻塞性肺疾病（chronic obstructive pulmonary disease, COPD）的良好指标。研究发现肺癌患者术前呼气峰值流量（peak expiratory flow, PEF）与术后发生痰潴留和肺部感染的相关性强，优于FEV_1_；进一步研究表明男性患者术前PEF＜320 L/min或女性PEF＜280 L/min或爬楼梯训练前后PEF值下降大于15%的患者，术后肺部感染发生率显著提高；因此，PEF改善或提高的程度就具有精准的数值，其评价的客观性和可操作性就高，临床易于应用和评估^[3, 31-33]^。另外，ERAS评价指标的客观性也体现在不同单位应用时受主观性因素的影响较小，如不同单位用不同的仪器或患者自身操作等。如FEV_1_可能受检测设备影响小，但不同的患者因对仪器使用方法及配合程度不同，肺功能室医生的讲解水平等，均影响FEV_1_数值的准确程度。从而导致临床差异，使不同单位研究的可重复性差，也导致了ERAS方案的优势不能充分显现或失败的主要因素。因此，我们目前的工作就应该通过临床研究并发现客观、精确的评价指标。

## 4 ERAS团队建设

"以问题为导向"是加速康复外科流程优化的关键，而"以病人为中心"则是ERAS的核心所在。问题导向的团队建设才能保障ERSA方案顺利的实施，根据ERAS方案实施过程中的问题，联系相关科室组成"虚拟中心"建立团队，并对团队进行统一培训，实时发现问题并处理，才能保证每个过程的顺利、有效的实施和优化。从目前研究看，ERAS方案核心及关键所在是观念更新，术前强调预防及处理（高危因素患者）、术中着重优化（传统方法）和术后管理（症状）。我们建议的是以问题立项目、以项目建团队，以团队订流程，以流程立方案，多中心求证据。从不断的临床实践中，探索合理，精准和有效的ERAS方案。

总之，肺癌患者ERAS方案的精准实施需要在ERAS方案实施的前、中、后均有正确的评估体系、合理的操作体系和客观的评价体系，才能保障ERAS方案的正确、有效执行，才能使进入流程的患者获得最大好处（表 1）。ERAS的理念只有贯穿于ERAS方案的各个流程才能达到理想效果，也只有准确的评估与评价才能不断优化流程和操作方案，从而使ERAS效益最大化。"可操作、可评估和可重复"的ERAS方案不但是我们研究的动力和方向，也是顺利推广和造福患者的唯一途径。

**1:** 肺癌患者外科ERAS体系 The enhanced recovery after surgery system for lung cancer

Assessment system	Operating system		Evaluating indicators	Goals
	Core	Key techniques		
Normal populations	Pathway optimization	Catheter management (Trachea cannula, urinary catheter, chest tube)	Length of stay (d)	Improve social satisfaction & decrease medical costs
Symptomatic populations	Symptom control	Pulmonary rehabilitation (Shortness of breath, exhaustion) Medication (Cough, pain)	Symptom control & quality of life	
High-risk populations	Pulmonary rehabilitation	Pulmonary rehabilitation Drug intervention Physical training	Decrease postoperative complications	
ERAS: enhanced recovery after surgery.				
